# Modeling Human Muscular Dystrophies in Zebrafish: Mutant Lines, Transgenic Fluorescent Biosensors, and Phenotyping Assays

**DOI:** 10.3390/ijms24098314

**Published:** 2023-05-05

**Authors:** Chiara Tesoriero, Francesca Greco, Elena Cannone, Francesco Ghirotto, Nicola Facchinello, Marco Schiavone, Andrea Vettori

**Affiliations:** 1 Department of Biotechnology, University of Verona, 37134 Verona, Italy; chiara.tesoriero@univr.it (C.T.); francesca.greco_02@univr.it (F.G.); francesco.ghirotto@univr.it (F.G.); andrea.vettori@univr.it (A.V.); 2 Department of Molecular and Translational Medicine, University of Brescia, 25123 Brescia, Italy; e.cannone@studenti.unibs.it; 3 Neuroscience Institute, Italian National Research Council (CNR), 35131 Padua, Italy

**Keywords:** muscular dystrophies, zebrafish, transgenic biosensors, disease phenotype, in vivo analysis

## Abstract

Muscular dystrophies (MDs) are a heterogeneous group of myopathies characterized by progressive muscle weakness leading to death from heart or respiratory failure. MDs are caused by mutations in genes involved in both the development and organization of muscle fibers. Several animal models harboring mutations in MD-associated genes have been developed so far. Together with rodents, the zebrafish is one of the most popular animal models used to reproduce MDs because of the high level of sequence homology with the human genome and its genetic manipulability. This review describes the most important zebrafish mutant models of MD and the most advanced tools used to generate and characterize all these valuable transgenic lines. Zebrafish models of MDs have been generated by introducing mutations to muscle-specific genes with different genetic techniques, such as (i) N-ethyl-N-nitrosourea (ENU) treatment, (ii) the injection of specific morpholino, (iii) tol2-based transgenesis, (iv) TALEN, (v) and CRISPR/Cas9 technology. All these models are extensively used either to study muscle development and function or understand the pathogenetic mechanisms of MDs. Several tools have also been developed to characterize these zebrafish models by checking (i) motor behavior, (ii) muscle fiber structure, (iii) oxidative stress, and (iv) mitochondrial function and dynamics. Further, living biosensor models, based on the expression of fluorescent reporter proteins under the control of muscle-specific promoters or responsive elements, have been revealed to be powerful tools to follow molecular dynamics at the level of a single muscle fiber. Thus, zebrafish models of MDs can also be a powerful tool to search for new drugs or gene therapies able to block or slow down disease progression.

## 1. Introduction

Muscular dystrophies (MDs) are a heterogeneous group of incurable hereditary diseases characterized by progressive weakness and the degeneration of skeletal muscle, leading to loss of ambulation, difficulties in the satisfaction of vital needs (i.e., breathing and eating), and death due to heart or respiratory failure [1]. 

Historically, MDs have been grouped according to the age of onset, the clinical and pathological manifestations, the pattern of inheritance (i.e., dominant, recessive, or sporadic due to de novo mutations), or the genetic causes as soon as they became clear [2]. MD subtypes such as laminopathies, sarcoglycanopathies, and dystroglycanopathies are, therefore, identified to underline the putative defective gene. However, such a classification, which is one of the most used, may result in some overlapping. Indeed, the same allelic mutations may give rise to a spectrum of clinical manifestations, and more than one gene may cause the same MD phenotype [2]. To further complicate the situation, the mutation of some MD-correlated genes has also been linked to diseases that do not primarily affect skeletal muscle [3,4,5]. In this review, the tremendous range of MDs was alternatively organized according to the localization/role of the mutant proteins that, over time, have been found to be disease-related. For example, the structural proteins of the basement membrane and extracellular matrix are distinguished from cytosolic proteins as well as the proteins involved in ER, and Golgi apparatus trafficking will be discussed separately from proteins involved in post-translational modifications. 

Progress in understanding the pathogenetic mechanisms of MDs has been achieved thanks to several well-established experimental models. In this review, we provide a complete overview of the MD animal models generated in zebrafish (*Danio rerio*) so far. 

Since the early 1980s, the zebrafish has emerged as a particularly valuable model organism [6,7] because of its unique advantages: (i) a small size and short generation time (adulthood is achieved in 3 months), (ii) the ease of husbandry and breeding, (iii) its high spawning productivity (up to 300 eggs once a week from a single mating event), (iv) its rapid and ex utero development, (v) and the optical clarity of the embryos and early larvae [8], as well as its genetic manipulability (see Section 4). The coexistence of the latter features makes this vertebrate organism particularly useful in the investigation of muscle functioning. In zebrafish, the skeletal muscle, which constitutes the main fraction of the body, becomes functional by the end of the first day of development, and it is fully differentiated after 48 h post-fertilization (hpf). The introduction of fluorescent transgenic tags may help to follow muscle development in living embryos via time-lapse confocal microscopy in a non-invasive manner [9]. Furthermore, as described in Section 1.1, zebrafish and human skeletal muscle show similarities in myofibrillar structure, contractile properties, and types of muscle fibers (fast and slow). Of course, the two species are evolutionarily far from each other, and they differ in several physiological and anatomical characteristics. For example, fast and slow muscle fibers in zebrafish are topographically separated, while they are mixed in humans. Additional disadvantages are the polyploidy of zebrafish for specific genes and a still limited number of cross-reacting antibodies. Despite these intrinsic limitations, the large amount of progress made over the last 20 years has demonstrated the usefulness of the zebrafish model in studying different aspects of human muscle diseases.

### 1.1. Skeletal Muscle Organization in Zebrafish

**Figure 1:**
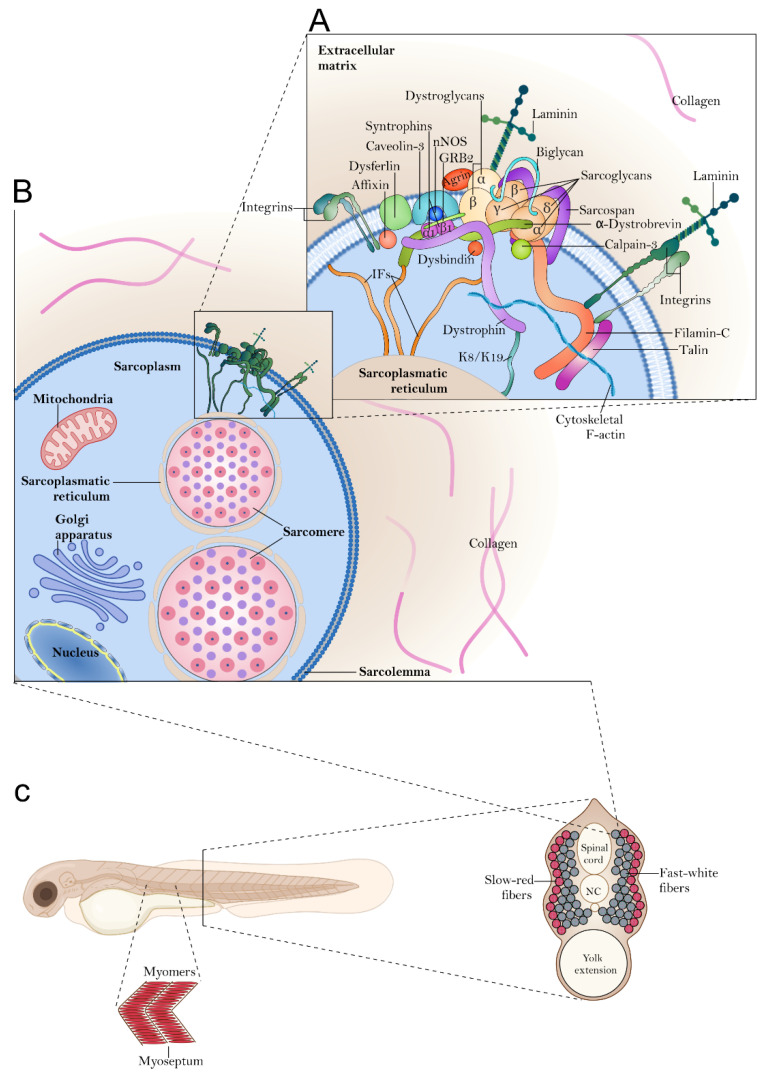
Zebrafish muscle organization. As teleost fish, zebrafish are characterized by two distinct types of muscle fibers: slow-red (in red) and fast-white muscle (in gray) (panel **C**). Regardless of the type, zebrafish skeletal muscle fibers are organized into myofibrils, which are long protein bundles surrounded by the so-called sarcoplasm and held together by the sarcolemma (panel **B**). Several proteins are involved in the functioning and maintenance of myofibril integrity. The (panel **A**) shows a model of the spatial configuration of the Dystrophin-associated glycoprotein complex (DGC) and related proteins that provide the connection of the sarcolemma to the basal lamina. In this panel is shown the dystrophin core complex (dystrophin, dystroglycans, sarcoglycans, sarcospan, syntrophins and dystrobrevins) as well as other DCG-associated proteins that forms a network with the extracellular matrix, the cytoskeleton, the sarcolemma, and the sarcomere. Because of graphical limitations, the physiological proportions of both cytoskeletal filaments and single proteins are not represented here. MD-associated genes encoding the (i) basal membrane and extracellular matrix proteins (ii) cytosolic proteins, (iii) dystroglycan and α-DG glycosylation-related proteins, (iv) nuclear envelope and cytoskeleton proteins, and (v) membrane proteins are listed in Tables reported below in the Chapter 2 of this review. Abbreviation: IFs, intermediate filaments. Image was partially created with BioRender (www.biorender.com), accessed on 2 March 2023.

MDs are characterized by the progressive degeneration of skeletal muscle caused by gene mutations affecting muscle fiber organization and function. Some of them have been studied in experimental zebrafish models. Before going into the details of zebrafish MD models, a brief description of the skeletal muscle structure is necessary to clarify the role of some proteins mentioned in the following sections. The correct organization of the skeletal muscle plays a critical role in both humans and zebrafish, as it serves many purposes. It allows for movement through the contractile system, sustains the body’s architecture, maintains body temperature, stores nutrients, and stabilizes joints. In humans, skeletal muscle is a highly organized tissue composed of bundles of fascicles formed by groups of muscle fibers that contain several myofibrils composed of actin and myosin proteins. Each myofiber represents a unique multinucleated muscle cell along with its functional unit, the sarcomere. Myofibers are closed by the sarcolemma, which has a polysaccharide coating that is fused to tendon fibers that anchor myofibers to the tip of the myotendinous junction [10]. In zebrafish, skeletal muscle fibers are also organized into sarcomeres and myofibrils, but they are segmented by myosepta throughout the body into myomeres, which are morphological functional units [11,12,13] (Figure 1). The myoseptum provides structural support to the zebrafish skeletal muscle and facilitates the rapid contractions necessary for zebrafish swimming [14]. Teleost fish possess two distinct types of muscle fibers, which include slow-red and fast-white muscles (Figure 1). The slow muscle fibers can typically be identified in the narrow V-shaped area near the lateral line and are generally used for long and continuous swimming activities. These fibers are characterized by a relatively small diameter, aerobic metabolism, and an abundance of mitochondria and glycogen. Fast muscle fibers constitute the majority of skeletal muscle in teleost fish, and their fiber diameter is comparatively larger than the slow ones. These fast-twitching fibers are primarily utilized for high-speed swimming activities such as predation and escape [15]. The muscle sarcomere is the basic unit of muscle contraction and is responsible for the generation of force and movement. The organization of the sarcomere is highly complex, and it is regulated by a large number of proteins that are essential for its proper function. The main proteins responsible for force generation during muscle activity are myosin, actin, troponin, and tropomyosin. Myosin is a motor protein responsible for muscle contraction. It is the most abundant protein of the muscle myofibrils and, with actin, forms the basic contractile unit known as the actomyosin complex. Another major protein in muscle fibers is tropomyosin (TPM), a protein that regulates the interaction between actin and myosin, thus controlling muscle contraction [16]. In zebrafish, there are six *tpm* genes that include the human paralogs of the *TPM1* (*tpm*1-1 and *tpm*1-2), the paralogs of the *TPM4* gene (*tpm*4-1 and *tpm*4-2), and the two single-copy genes *TPM2* and *TPM3* [17]. Another structure that plays a major role in maintaining sarcolemmal integrity and function is the dystrophin-associated glycoprotein complex (DGC) (Figure 1). This protein structure connects the cytoskeleton to the extracellular matrix (ECM) and is composed of numerous cytoplasmic proteins such as dystrophin, syntrophin, and dystrobrevin, as well as transmembrane proteins such as dystroglycan, sarcoglycan, and sarcospan [18] (Figure 1). Outside the myofiber, laminin-2 binds to the DGC through α-dystroglycan, thus linking the sarcolemma to the ECM (Figure 1). Similarly, on the cytoplasmic side, dystrophin binds to F-actin and β-dystroglycan (Figure 1). In turn, α- and β-dystroglycans, which are closely associated, form a stable membrane complex, providing additional structural support to the DGC that is essential for transmitting force and stabilizing the membrane during contraction [19]. Losing DGC structure results in the disruption of sarcolemma upon mechanical stress, and it is not surprising that mutations occurring in DGC-associated genes are often linked to the onset of severe forms of MD. Mutations in the *DMD* gene, which encodes dystrophin, cause Duchenne muscular dystrophy (DMD) and Becker muscular dystrophy (BMD), one of the most studied forms of MD. Moreover, mutations in genes encoding for dystroglycan, such as *DAG1* or Fukutin-related protein (*FKRP*), cause various forms of MD, including muscular dystrophy–dystroglycanopathy type A9 (MDDGA9) or limb–girdle muscular dystrophy (LGMD) type 2I [20,21,22]. Mutations occurring in the genes that encode sarcoglycans can be associated with different forms of LGMD, such as LGMD2C, LGMD2D, LGMD2E, and LGMD2F [23]. The loss of sarcoglycans destabilizes the dystrophin complex, increasing susceptibility to mechanical stress and damage to muscle fibers. Finally, in addition to the DGC core proteins, the disruption of other molecules can be also associated with MDs. These proteins include the matrix protein laminin A2, which is linked to congenital MDs [24]; the protein telethonin, associated with LGMD2G [25]; or the transmembrane protein dysferlin, which plays a role in muscle membrane repair and regeneration. Mutations in the *DYSF* gene, which encodes dysferlin, can cause several types of MD, including LGMD2B [26]. Moreover, extracellular proteins such as collagen VI, the calcium-activated protease calpain, and the intermediate filament protein lamin A/C, have also been associated with the onset of Ullrich/Bethlem congenital muscular dystrophy [27], LGMD2A [28], and Emerye–Dreifuss/LGMD1B dystrophy, respectively [29]. In conclusion, the proteins involved in muscular dystrophy play essential roles in maintaining the structure and function of muscle cells. Mutations in genes encoding these proteins lead to the loss of stability and integrity in the muscle cell membrane, making the muscle fibers more susceptible to damage and degeneration. Understanding both the molecular mechanisms underlying the total or partial absence of these proteins and their main interactions is critical to developing effective therapies for MDs.

## 2. Models of Muscular Dystrophy in Zebrafish

### 2.1. Basal Membrane and Extracellular Matrix Protein

The ECM comprises 1–10% of the total muscular weight, depending on the skeletal muscle, and it has several fundamental functions such as force transmission, support, and the repair of muscle fibers after damage. Moreover, the ECM provides a particular architecture that maintains myofibers, blood vessels, and nerves in close contact inside the muscle [30]. In addition, the composition of the ECM may influence the contractile properties and the gene expression of the muscular fibers, changing the rigidity of the basal membrane [31]. Due to all these characteristics, alteration in the composition and components of the ECM can lead to the onset of several pathologies, including MDs.

As described previously, one of the most important EMC components is laminin α2, which is encoded by *LAMA2*. In humans, mutations of this gene can give rise to the development of a range of MD spectra, from the more frequent and aggressive Merosin-deficient congenital muscular dystrophy 1A (MDC1A) to a milder pathology reassembling the LGMD phenotype with later-childhood onset [32]. The dystrophic zebrafish mutant *candyfloss* (*caf^teg15a^*), which is generated by N-ethyl-N-nitrosourea (ENU) mutagenesis, shows non-sense mutations in the globular domain of the laminin α2, mimicking the clinical genetic lesions of MDC1A patients [8]. Interestingly, *candyfloss* mutants display muscle fiber detachment from the myotendinous junction (MTJ) starting by 72 hpf, with muscle degeneration and, consequently, altered motility as well as fewer coils [8,33]. In addition, homozygous mutants in most cases present death by 16 days post-fertilization (dpf) and a very small or absent production of progeny [8]. Moreover, muscle fiber detachment is induced by motor activity [8], in which the mechanical overload of mutant muscles increases the severity and progression of the pathology, though embryo immobilization can salvage the disease phenotype [34]. However, unlike other MD models, such as *sapje* (dmd^tm90c^) fish, where the dystrophic fibers are short-lived due to sarcolemma disruption, in the *candyfloss* mutant, the muscle fiber detachment does not affect sarcolemma integrity, and the myofibers live longer [35,36]. The *candyfloss* muscle fibers show the ability to undergo cellular remodeling, in particular forming membrane protrusions and branches [35]. Moreover, after detachment, the myofibers go through a process of fusion, thus demonstrating that several survival mechanisms are activated in post-detachment muscle fibers. Importantly, such behavior seems to be specific to LAMA2 dystrophic fibers, since dystrophin mutant myofibers do not display any of these features [35]. Furthermore, *candyfloss* myofibers present an abnormal lysosome distribution at the sarcolemma, in which these organelles can be involved in mechanisms of membrane repair and the removal of cellular debris after detachment, though further evidence is needed to understand the specific function of this phenomenon [37]. Autophagy does not appear to be the only altered cellular process, considering that the proteasome degradation pathway seems to be upregulated in different types of MD, including MDC1A. Overexpression of the proteasome complex and an increase in protein ubiquitination can be used as dystrophies hallmarks and inhibition of proteasome activity with the MG-132 inhibitor can rescue the pathological phenotype [37].

*Candyfloss* is not the only zebrafish model for the diseases related to *LAMA2* mutations, but another zebrafish line, called *lama2^cl501^*, has been identified through ENU mutagenesis and further analyzed. The *lama2^cl501^* mutant contains a mutation in the coiled-coil domain in the long arm of LAMA2, thus resulting in the loss of interaction between laminin α2 and the other two laminins of the heterotrimeric complex [38]. The phenotype of this mutant overlaps with that of the *candyfloss* zebrafish, in which the main pathological manifestation is the detachment of myofibers from the MTJ without disrupting the sarcolemma. Moreover, the *lama2^cl501^* mutant displays other phenotypic features, such as lower laminin α2 expression in the basal membrane, smaller myotomes, defects in the sarcomere architecture, and brain and eye alterations [38].

Although laminin α2 is one of the major components that maintain attached muscle fibers, other laminins may be involved in anchoring myofibers to the MTJ, since injecting the *LAMA1* morpholino into *LAMA2* mutants results in a more severe phenotype [39]. This is also suggested by the fact that the *ilk/dmd* and *ilk/dag1* double-homozygous mutants present a more severe disease than the *LAMA2/dmd* and *LAMA2/dag1* mutants. This also demonstrates that proteins directly or indirectly interacting with laminin α2, such as dystroglycan, dystrophin, and ilk, if altered, can modify the *LAMA2*-MD phenotype [39,40]. 

As outlined above, collagens also have different fundamental functions such as providing elasticity, support, and organization to the ECM. In particular, collagens are essential in forming the MTJ and play a major role in keeping myofibers attached to the ECM [41]. For this reason, the different zebrafish models generated with morpholine, TALEN, or CRISPR/Cas9 mutagenesis display similar features (see Table 1). Mutations in one of the three genes that codify the components of collagen VI lead to the onset of either Ullrich congenital MD (UCMD) or Bethlem myopathy (BM) [42], while alterations in the *col22a1* gene can cause disruption in the MTJ [43]. In particular, the different zebrafish lines exhibit alterations in the organization of slow muscles and the ECM throughout the different stages of their life cycles. This phenomenon becomes more evident during mechanical stress, thus inducing the detachment of the myofibers from the MTJ, reassembling a phenotype similar to the *candyfloss* model [44]. Moreover, both the morphants and mutants show abnormal mitochondria, an abnormal endoplasmic reticulum (ER), defective autophagy, and fibrosis [44,45,46]. Interestingly, as the *col6a1^ama605003^* mutant ages, it develops a stress behavior due to hypoxia, thus leading to several respiratory-adaptive responses such as residing in a surface area with a higher concentration of oxygen and increasing the respiratory rate [45].

### 2.2. Cytosolic Proteins

Dystrophin was the first protein identified as causative of MD. It localizes in the cytosol, although it is directly related to the muscle sarcolemma as a founder of the dystrophin-associated protein complex (DAPC). From its early discovery in the late 1980s, many mammalian and non-animal models have been generated. In 2002, Parsons et al. [48] described the first zebrafish model for skeletal MD, where the dystroglycan was knocked down by a morpholino oligonucleotide (MO), which created an MD phenotype. One year later, Bassett et al. [49] molecularly characterized the first stable zebrafish MD model, known as *sapje*. This mutant was earlier identified by Granato and colleagues [50] during a mobility dysfunction screening of zebrafish mutagens generated using ENU at Tübingen University. In particular, the mutation harbored by *sapje* led to reduced striation and somite degeneration; although the zebrafish embryos presented with muscle fiber formation comparable to the wild-type, at 96 hpf, the swimming velocity and muscle birefringence were reduced. During development, the lesions become severe in the somitic segments, and the degeneration was restricted to skeletal muscle tissue, but heart and jaw muscles were not affected. Basset et al. [49] described the degeneration mechanism of full *sapje* embryo mutants as the separation of skeletal muscle fibers from their attachment points on myosepta. Furthermore, they identified the *sapje* locus as *dmd* [51], nowadays well known as the *Danio rerio* ortholog of the *Homo sapiens DMD* gene, and this showed that the mutant carries a non-sense mutation in exon 4 of the zebrafish dystrophin gene. 

The characterization of the zebrafish *sapje* mutation revealed a novel pathological mechanism together with the validation of a model for DMD and BMD that is currently not only a worldwide landmark but also a valuable platform for compound screenings, thus allowing for new insights into a wide range of diverse muscle diseases. 

An additional zebrafish dystrophic mutant was also screened via the early-pressure technique and genetically isolated based on the symptoms of muscle disease presence by Guyon et al. [52]. They identified 8 out of 500 different mutants presenting abnormal birefringence, among which, one had a phenotype very similar to the *sapje* model, and it was thus called *sapje*-like. Like *sapje* zebrafish, the *sapje*-like model showed clear muscle degeneration at 5 dpf, and sequencing analysis showed that it carries a mutation in the donor splice junction of exon 62 of the dystrophin gene. These results represented the characterization of the first zebrafish model harboring a splice-site mutation in the dystrophin gene, exploitable for new studies and therapies for muscle disorders associated with splicing. 

Another cytosolic protein involved in the dystrophy scenario is *DUX4*. Its role in late-onset facioscapulohumeral dystrophy type 1 (FSHD-1), which is the most common autosomal dominant form of MD, was described by Kunkel’s research team, who generated a *dux4* injection model (DUX4i) [53] and a *dux4* transgenic model (DUX4t) [54]. In particular, despite the FSHD-1 onset in the second decade of life, *DUX4* misexpression triggers muscle degeneration in the early stages and, therefore, it appeared to have a development role rather than being causative. During the larval period (3–5 dpf), the two mutants presented abnormal birefringence and late hatching; the DUX4i also showed abnormal eye and ear formation, together with fin asymmetry, while DUX4t swam shorter distances and had a lower twitch and tetanic force. This phenotype was mild in adult DUX4i mutants, whose lipids and collagen were neutral together with inflammation and muscle function. They also showed fin and muscle regeneration. On the contrary, DUX4t mutants developed abnormal muscle, asymmetrical fat deposition, and collagen accumulation. Furthermore, muscle function was fully abnormal. The DUX4 developmental role was also confirmed by Wallace et al. [55], who over-expressed the gene in zebrafish embryos. At 4 dpf, the mutants showed defects in muscle histology, curved bodies, fin abnormalities, and cardiac hypertrophy.

Particularly noteworthy are the stable muscleblind-like (*mbnl*) zebrafish mutants generated by Hinman and colleagues to study the pathogenetic mechanisms of myotonic dystrophy [56]. The MBNL family is composed of three RNA-binding proteins (MBNL1, MBNL2, and MBNL3), which are highly conserved across several species. In zebrafish, loss-of-function mutations associated with these proteins lead to physical and molecular alterations consistent with those present in myotonic dystrophy patients with mutations in the *DMPK* and *ZNF9* genes. Interestingly, single, double, and triple *mbnl* homozygous zebrafish showed a relevant disease phenotype, including impaired motor behavior and decreased body size [56]. Transient *mbnl* zebrafish models were also generated [57,58], but their characterization pointed out several limitations compared with the stable mutant models [56].

Moreover, two CRISPR/Cas9 zebrafish KO models for the Valosin-containing protein (*vcp)* gene were recently generated and validated for mechano-pathological studies of VSPopathies. VCP is a type II AAA ATPase involved in cellular protein homeostasis that has been associated with the onset of IBMPFD syndrome and some rare variants of ALS or myofibrillar myopathy. In two recent works, the 72 hpf old KO crispants of the gene showed (i) a reduction in the birefringence signal, (ii) disorganization in both myofibrils and sarcomeres, (iii) dysmorphic mitochondria, and (iv) a significant reduction in the distance moved after a touch-evoked escape response test [59,60]. The phenotype of the *vcp* crispants, also showing defects in cardiomyocyte functions and disruption in protein homeostasis, recapitulated skeletal muscle myopathy, which is consistent with previous findings and previous morpholino-mediated *vcp* knockdowns [59,60,61].

The disruption of many other cytosolic proteins has been associated with the onset of different dystrophy forms, but only transient zebrafish models have been described, as listed in the following Table 2. 

### 2.3. Dystroglycan and α-DG Glycosylation-Related Protein

As described in Section 1.1, dystroglycan (DG) is an essential and conserved DAPC component that plays both a structural and functional role. It is encoded by the homonymous gene (*DAG1*) and consists of two subunits, the transmembrane β-DG and the extracellular α-DG, which requires complex post-translational modifications, particularly glycosylation, by a group of enzymes to exert its ligand-binding function [73].

DG impairment has been associated with a variety of MDs, generically known as dystroglycanopathies (DGPs). In particular, mutations of the glycoprotein DG have been grouped as primary DGPs, whereas secondary DGPs are caused by defective DG glycosylation. Nowadays, about 20 genes have been linked with secondary DGP [32]. Since their mutation is often associated with different phenotypes, it is becoming clearer that the disease severity is instead largely correlated with the level of α-DG hypo-glycosylation, and the upregulation/rescue of a single enzyme could be beneficial for multiple forms [74]. 

The first zebrafish model of DGP was described by Parsons and colleagues [48], who used an antisense morpholino oligonucleotide (MO) to knock down DG. The resulting morphant embryos were characterized by delayed development together with a hooked tail, less flexibility, uncoordinated movement, and compromised muscle integrity [48]. In zebrafish, the complete absence of both the α- and β-DG subunits was subsequently achieved via ENU mutagenesis screening [75]. The resulting model system, named the *pathchytail* (*dag1^cl500^*) mutant, was characterized by a late onset of the MD phenotype, with muscle birefringence defects becoming evident at 7 dpf, as well as disorganized T-tubules. Moreover, brain abnormalities and ocular defects have commonly been observed in *pathchytail* mutants, suggesting additional roles for DG in brain development [75]. 

As mentioned before, secondary DGPs are caused by defects in α-DG glycosylation, a modification necessary to properly perform its function. Over the years, several dystrophic-like zebrafish models have been generated by the morpholino-mediated knockdown of orthologous genes encoding for enzymes whose mutations are associated with secondary DGPs in mammals. The down-regulation of these proteins results in fish showing many defects, such as muscular disorganization, severe neurological and ophthalmic impairments, and often early death—for more details, see Table 3. Particularly noteworthy are stable *fkrp* zebrafish mutants, which were recently generated by using different genome editing strategies [76,77]. The *fkrp* zebrafish mutant, generated by Serafini et al. [76], phenocopies the symptoms observed in patients affected by Walker–Warburg syndrome, such as muscle breakdowns, head malformations, and early death. Thus, it could be a useful tool for future high-throughput drug screenings to identify new therapeutical compounds for MD treatments. Moreover, a second *fkrp* zebrafish mutant revealed, for the first time, that *fkrp* is also involved in fibronectin sialylation occurring in the Golgi, an essential process both for fibronectin–collagen binding and for the maintenance of the muscle basement membrane integrity [77]. Such observations may be a possible explanation for the wide spectrum of DGP clinical manifestations.

### 2.4. Nuclear Envelope and Cytoskeleton Proteins

The skeletal muscle cytoskeleton is organized in a complex way to coordinate muscle contractions. The fundamental unit is the sarcomere, composed of several structural and regulatory proteins, for which interaction with intermediate filaments is necessary both for the maintenance of cellular integrity and for the transduction of mechanical stimuli. Further, the intermediate filament lattice links the entire contractile apparatus to the sarcolemma and other organelles, such as the nucleus, mitochondria, lysosomes, and the sarcoplasmic reticulum [92]. The intermediate filament (IF) network is mainly composed of the muscle-specific IF protein desmin, which is associated with other IF proteins, such as synemin, paranemin, and syncoilin, as well as lower amounts of keratins K8 and K19 [92]. Desmin and its associated proteins form a network around the Z-disks, connecting them to each other and to the sarcolemma [93]. Except for paranemin, all the IF proteins are associated with costameres. This association is mediated by dystrobrevin (through synemin and syncoilin), dystrophin (through synemin and keratin 19), and myospryn. Keratin filaments (K8/K19) localize around the M-lines and near the cell surface, where they also link to costameres. The complex network of the IF lattice also provides effective functional crosstalk between organelles, such as the ER and mitochondria, and promotes lipid and calcium traffic. Thus, it is not surprising that alterations to the integrity of the IF network might lead to cardiac or skeletal myopathy [92].

Mutations in the desmin (*DES)* gene lead to myofibrillar myopathies, a group of muscle disorders characterized by the presence of heterogeneous desmin protein aggregates [94]. Paulin and colleagues provided novel insights into the link between aggregate deposition and myofibrillar degeneration [95] by generating two zebrafish knock-in lines named *desma^Ct122aGt^* and *desma^Ct122aRGt^*. These two mutant lines revealed both that the protein aggregation does not simply depend on specific mutations occurring in the *desma* gene and that protein aggregate deposition leads to myofibrillar alterations similar to those observed in the KO *desma^sa5−/−^* zebrafish mutant line, previously obtained via ENU mutagenesis screening. It also presented perturbed organization in the myofibrils, together with a decreased swimming capacity [94]. Similar skeletal muscle phenotypes were also observed in desma morphants, together with a reduction in the hatching rate, spontaneous movements, body length, time, and distance moved, as described in Li et al., 2013 [96] and Bührdel et al., 2015 [61]. On the contrary, knocking down the zebrafish *desmin* genes using CRISPR/Cas9 did not lead to skeletal muscle degeneration but altered calcium flux in myofibers [97].

Nicolas and colleagues [98] described the first transgenic zebrafish model for the laminin A/C gene, generated by using the CRISPR/Cas9 mutagenesis system. Zebrafish *lmna* mutants display alterations in skeletal muscle organization, transient cardiac abnormalities, and anomalous motor behavior; in fact, the mutants swim less and slower than normal, mimicking the Emery–Dreifuss muscular dystrophy (EDMD) phenotype. Indeed, both homozygous and heterozygous mutants show skeletal muscle damage, with an irregular distance between the myofibers and cytoskeletal modifications due to the disruption of F-actin, which can no longer interact with laminin-A/C, thus destabilizing the nucleo-cytoskeletal architecture and impairing the response to mechanical stimulus. Moreover, the upregulation of phosphorylated Pkc α and Erk 1-2, as well as changes in skeletal-muscle-gene-related expression (e.g., aberrant MAP kinase signaling pathway activation), were observed in both crispant and morphant zebrafish models for laminin-A/C [98]. 

Another interesting zebrafish model is the *stretched-out* (*sot^p1cpej^*) mutant, characterized by a genetic lesion of the *flncb* gene that is associated with the early onset of a severe form of myofibrillar myopathy. This mutant shows damaged or disrupted slow muscle fibers but no alteration in fast fibers at 26 hpf. Further analysis revealed that embryos at 32 or 48 hpf did not present any alteration, thus suggesting that defective fibers can go through turnover and can be replaced by new slow muscle fibers [99]. Indeed, the turnover and replacement of fibers in the *sot* mutant may be due to a change in gene expression, from *flncb* to *flnca*, at the level of the primary slow muscle fibers. This indicates that the two gene isoforms are functionally redundant and that the total level of Flnc expression is crucial to fiber maintenance. This is also demonstrated by the fact that the loss of *flnca* causes a mild phenotype very similar to that of the *sot* mutant, whereas the loss of both isoforms leads to weakness and the almost total loss of slow muscle fibers [100]. 

Other nuclear and cytoskeletal components are involved in the development of a wide spectrum of MDs, whose models are summarized in Table 4.

### 2.5. Membrane Proteins

Caveolin-3 is one of the major integral membrane proteins related to caveolae, small invaginations of the plasma membrane abundant in animal cells [106]. It is mainly expressed in cardiac and skeletal muscle cells, and mutations in the caveolin-3 gene (*cav3*) have been identified in human patients with different muscle diseases, including LGMD [107]. 

Several zebrafish models have been generated for *cav3* using techniques such as mRNA injection, morpholinos, and the Tol2 transposon system [63,108]. The resulting mutants display large intercellular space and shorter mononucleated myofibrils, showing oval and ramified shapes as well. Moreover, morphants show disorganized contractile apparatuses, abnormal endomembrane systems, and the upregulation of *eng1a* gene expression, all of which induce an increase in muscle progenitors neighboring the notochord, thus demonstrating the main role of caveolin-3 in muscle maintenance and fiber fusion [108]. Interestingly, the transgenic *cav3* dystrophic mutant shows membrane disruption but does not show evidence of fiber detachment like in the *candyfloss* and *sapje* mutants [8,49], suggesting another mechanism leading to membrane damage. The consequent weakness in the sarcolemma may trigger apoptosis, demonstrating the importance of caveolin-3 for membrane integrity, especially during muscular activity [63]. 

Furthermore, zebrafish morphant models have also been generated for other membrane proteins, resulting in skeletal muscle damage and disorganization as well as the abnormal development of the eyes and brain (see Table 5).

## 3. Functional and Genetic Tools for Investigating Skeletal Muscle Development and Functions

All previously described zebrafish models of myopathies are useful tools to study these disorders, as they share similar features with human diseases. Most of them can be easily characterized using many functional, behavioral, and phenotypical assays to test motor behavior, muscle force, muscle structure, calcium homeostasis, oxidative stress, mitochondrial function, and metabolism.

### 3.1. Motor Behavior

Several methods are available to assess motor behavior in zebrafish at different stages of development. Zebrafish embryos, larvae, and adults show different stereotyped motor behaviors depending on both morphological and functional changes in cells related to locomotion, such as muscle fibers, motor neurons, and cells involved in the development of the swim bladder.

At 17 hpf, the time when muscles start to be innervated by primary motor neurons, spontaneous coiling contractions are observed, which reach a peak of frequency within 3 h and progressively decline until 26 hpf [112].

By 27 hpf, embryos start to respond to mechanical stimuli, swimming around a Petri dish if touched with a little tip near the nerve centers [46]. More mature swimming, linked to beat-and-glide responses, appears around 5 dpf.

In this context, (i) spontaneous coiling events or tail flips (to assess side-to-side coiling contractions) [113], (ii) responses to external tactile stimuli (touch-evoked escape response test) [114], (iii) and swimming motor behavior (Noldus DanioVision System recording), have been extensively described and used through the years to evaluate dystrophic lines [46,115,116,117,118,119].

Spontaneous coiling contractions can be recorded with light microscopy as the number of events observed in 15 s or more for individual embryos.

The touch-evoked escape response test is performed by touching embryos and evaluating their ability to escape after a stimulus. This test is frequently measured using a scale of four values: 3, normal motor behavior; 2, mild motor impairment; 1, coiling events or flickers of movement both related to a severe motor impairment; 0, complete paralysis [46,119]. In addition, the measurement of these two assays can be automated [120].

Zebrafish motor behavior comprises a complex repertoire within a window between 17 and 120 hpf. It can be analyzed using different video tracking hardware and software, which provide readouts on zebrafish behavioral responses to a great variety of stimuli: light/dark, acoustical, environmental, and compounds [121,122,123].

As proposed by Stocco et al. [118], zebrafish larvae can be stimulated to swim using a time routine, repeated several times, involving exposure to 5 min of light periods after 30 min of incubation in the dark. After the test, the total distance moved can be analyzed.

Recently, a swimming tunnel instrument was developed to measure different parameters linked to the motor behavior of both zebrafish larvae and adults. It consists of a glass tube, filled with fish water, connected to a specific instrument that measures parameters, such as distance moved, muscle force, oxygen consumption rate, and metabolic changes, which will be recorded on a computer equipped with specific software [124,125,126,127,128].

### 3.2. Muscle Force (Electrophysiology)

Muscle force in zebrafish larvae should be analyzed also using electrophysiology. Briefly, larvae between 48 and 120 hpf are anesthetized and decapitated. Then, the body trunk is transferred to an experimental chamber, and the sample’s proximal end is coupled to a titanium wire connected to an isometric force transducer. The distal end of the titanium wire is linked to a high-speed motor lever arm. Muscles are then stimulated with a biphasic muscle stimulator through platinum electrodes [129,130].

The electrophysiological method has been successfully applied to different zebrafish models of MD, which show a progressive reduction in contractile force during disease progression [130,131,132,133].

### 3.3. Muscle Structure

The fastest and most reliable method to assess muscle fiber structure in zebrafish is birefringence, a technique that measures muscle anisotropy. which is the ability of muscle fibers to refract polarized light [50,134]. This technique offers the possibility of identifying and quantifying muscle damage in zebrafish at 40 hpf in a less invasive way [110,135]. Birefringence is also applied routinely to perform high-throughput drug screening and test drug efficacy [136,137].

The technology most used to observe the detailed ultrastructure of muscle tissue is transmission electron microscopy (TEM). This high-performance technique offers the possibility of obtaining qualitative and quantitative data concerning sarcomeric organization, the basement membrane, the MTJ, and the myoseptum structure, together with mitochondrial alterations, such as outer mitochondrial membrane detachment and swelling; quantitative analyses of distances between the outer and inner mitochondrial membrane can be also performed [11,43,117,118].

The availability of transgenic zebrafish biosensors [138] and several specific fluorescent antibodies help visualize the structure of a single muscle fiber using the most sophisticated lightsheet, confocal, and two-photon microscopy. In particular, antibodies (as described in the protocol of Gody and Henry, 2013 [139]) have been used to visualize filamentous actin and changes in the organization of myofibers in several zebrafish dystrophic muscle models [33,77].

### 3.4. Calcium Homeostasis and Oxidative Stress (Use of Fluorescent Probes)

Several methods based on genetically encoded biosensors that could be directly injected into zebrafish embryos or integrated into the zebrafish genome are used to visualize tissue-specific metabolic changes. Genetically encoded biosensors (FiNad sensors) of a wide dynamic range can easily be used to track cytosolic NAD+ and NADH redox states in vivo [140,141]. Other specific biosensors, such as Laconic [142], could be useful in assessing in vivo metabolic changes by observing lactate concentrations in muscles. Recently, two other genetically encoded biosensors, Rogfp2-Orp1 and Grx1-Rogfp2, were developed to measure in vivo hydrogen peroxide levels and glutathione redox potential, respectively [143]. Zebrafish transgenic lines that specifically express these biosensors in the muscle fibers could be another useful tool to explore the redox state in living animals. The measurement of mitochondrial reactive oxygen species (ROS) can be also achieved using fluorescent probes, such as the mitoSOX probe, which is able to react with ROS derived by both hydrogen peroxide and oxygen, as in the protocol of Rissone and Candotti [118,144,145].

The dysregulation of Ca^2+^ ions in muscle fibers is a primary cause of fiber death. Thus, different genetically encoded calcium indicators have been developed to check both calcium levels and dynamics in vivo at the level of a single muscle fiber [146,147,148]. These indicators are based on modifications of calmodulin (CaM), in which the calcium-binding domain is fused to GFP. GCaMP indicators with different calcium sensitivities have been developed (GCaMP1, GcaMP3, GcaMP6, GcaMP6f, GcaMP6s, and GcaMP7) to check the cytoplasmic levels of calcium [147,149,150]. Modified GCaMP6f, which harbors a mitochondrial-specific leader peptide at the N-terminus, was developed to check calcium levels in mitochondria [151]. 

### 3.5. Mitochondrial and Metabolic Function

The mitochondrial functions of zebrafish models of MD can be assessed with several methods.

The most common assay to evaluate the mitochondrial membrane potential is the accumulation of the tetramethylrhodamine methyl ester (TMRM), a fluorescent potentiometric probe targeting the mitochondrial membrane. This assay can be performed by incubating zebrafish embryos at 24/48 hpf with 300 nM of TMRM and 1.6 μM of CsH (to inhibit the multiple drug resistance pumps) in the dark for 24 h [118].

A powerful technique to measure (i) mitochondrial and cell respiration through the oxygen consumption rate (OCR) and (ii) metabolic changes through the extracellular acidification rate (ECAR) is the Seahorse (Agilent Technology), which has been successfully applied to zebrafish, as demonstrated by Bond et al., 2018 [152]. All measurements can be performed on a single zebrafish sample (between 24 and 96 hpf) placed in the well of an islet capture microplate and treated sequentially with oligomycin, FCCP, rotenone, and antimycin A [117,118].

Mass spectrometry possesses great potential to characterize the proteome of zebrafish models of MD to identify both mechanisms involved in disease pathogenesis and new potential druggable targets [153]. It also offers the possibility of evaluating the efficacy and toxicity of potential drugs [154] and obtaining specific profiles of protein modifications, such as the glycome profile [155]. Mass spectrometry can be performed at all stages of zebrafish development, from embryos to adults, by isolating proteins from entire animals or specific tissues. 

## 4. Transgenic Fluorescent Zebrafish Lines

### 4.1. Zebrafish Reporter Lines Targeting In Vivo Signaling Pathway Activities Involved in Muscle Development

In this section, we provide a novel approach to system biology using transgenic biosensor animals that express fluorescent proteins under the control of signaling pathway-responsive cis-elements. 

Pathway-specific responsive/regulatory elements (REs) can be multimerized to increase the responsiveness of the intended reporter and possibly attract more signal-dependent transcription factors. With a minimal promoter and a polyA signal site, multimerized REs are cloned upstream of a reporter gene encoding a fluorescent protein [138].

Bone morphogenetic proteins (BMPs), the wingless-related integration site (Wnt), sonic hedgehog (Shh), the hypoxia-inducible factor (HIF), the Nrf2-ARE pathway (Nrf2/ARE), the glucocorticoid reporter (GRE-reporter), and cAMP-response element-binding protein (CREB) signaling are crucial in controlling cell proliferation, stem cell maintenance, and differentiation in skeletal muscle. Several transgenic zebrafish lines have been created to investigate the aforementioned signaling pathways and the effects of their hypo- or hyperactivation in skeletal muscle (Table 6).

The HIF reporter zebrafish, named *Tg(4xHRE-TATA: eGFP)^ia21^* [156], expresses tandem copies of a hypoxia response element (HRE), driving eGFP expression. The HIF pathway is active during zebrafish development, and transgenic embryos exhibit fluorescence from 24 hpf onward in muscle, lenses, skin, hearts, retinas, neurons, notochords, and the circulatory system.

The Shh signaling pathway has been demonstrated to be involved in skeletal muscle development in zebrafish [157]. To visualize in vivo Shh signaling with high detection sensitivity and a single-cell resolution, different transgenic zebrafish were generated carrying Gli-dependent fluorescent reporters. The Shh-pathway-driven expression of exogenous genes in cultured cells or live organisms was previously achieved using a minimal δ-crystallin promoter and eight tandem Gli binding sites derived from the murine *Fox2A* floor plate enhancer [158]. Mich et al. prepared reporter constructs that coupled these regulatory elements with sequences encoding nuclear-localized fluorescent proteins tagged with the ornithine decarboxylase-derived destabilizing peptide *Tg(8xGliBS:mCherry-NLS-Odc1)^st1002^*. Moreover, Corallo et al. generated transgenic lines that coupled these regulatory elements with sequences encoding localized fluorescent protein alone (eGFP or mCherry). The Shh reporter line shows fluorescence activity in the somitic region of a *Tg(8xGliBS:mCherry-NLS-Odc1)^st1002^* zebrafish since 24 hpf, allowing for the labeling of muscle progenitor cells and superficial slow-twitch muscle fibers [159,160].

Wnt/β-catenin signaling in live embryos can be studied using several transgenic lines. The fluorescent reporter in the *Tg(top:GFP)^w25^* transgenic line is under the control of TOPFlash, which consists of four consensus TCF/LEF binding sites placed next to a *c-fos* minimal promoter [161,162]. Additionally, transgenic fluorescent zebrafish lines, called *Tg(7xTCF-Xla.Siam:GFP)^ia4^* and *Tg(7xTCF-Xla.Siam:nlsmCherry)^ia5^*, which are driven by the enhancer region of the minimal promoter of the *Xenopus* gene *siamois*, with seven tandem repeats of the TCF/LEF binding elements, have been developed [163]. With these reporter lines, Moro et al. [163] identified zebrafish tissues sensitive to canonical Wnt stimuli, including larval brains, notochords, otic vesicles, somites, fin buds, eyes, and posterior lateral lines.

Pyati et al. [164] created a transgenic zebrafish line that had a dominant negative BMP receptor linked to GFP integrated into its genome under the control of the heat shock promoter *Tg(hsp70l:dnBmpr-GFP)*. By subjecting this transgenic line to heat shock at various embryonic stages, the researchers were able to distinguish between the functions of BMP signaling before and after mid-gastrulation [164]. Many transgenic lines have been created that show Smad-mediated BMP signaling in embryos and adults to visualize BMP signaling in live embryos: *Tg(hsp70l:dnBmpr-GFP)^w30^*, *Tg(BmpRE-AAVmlp:eGFP)^mw29^*, *Tg(BRE-AAVmlp:d2GFP)^mw30^*, *Tg(BRE-AAVmlp:dmKO2)^mw40^*, *Tg(BMPRE:mRFP)*^cj100^, and *Tg(BMPRE:NLS-mCherry)^ia17^*. These lines express eGFP, destabilized eGFP, destabilized KO2, or nuclear mCherry under the well-characterized BMP-responsive element (BRE), adopted from the mouse inhibitor of the differentiation-1 enhancer. The developing tailbud, hematopoietic lineage, dorsal eye, brain structures, heart, jaw, fins, and somite muscle, as well as other tissues, were found to dynamically express these fluorescent proteins [165,166,167,168].

To visualize CREB Signaling, Giuliodori et al. [169] generated a reporter line with a multimerized CREB-responsive element (6XCRE: 6X cAMP-responsive elements, 5′- TGACGTCA -3′). The transgenic reporter line is responsive through the transcription-factor-mediated activation of the regulatory elements located upstream of the coding sequences of the reporter protein [143]. Specifically, this reporter line shows CREB signaling activation in the heart and skeletal muscle, as well as in the nervous, hematopoietic, and endocrine systems (N. Tiso, personal communications).

Glucocorticoids (GCs) regulate many cellular processes including skeletal muscle development through the binding of the glucocorticoid receptor (GR) to specific REs located upstream of the transcription starting site or within an intron of GC target genes. The Dalla Valle group generated the transgenic line *Tg(9xGCRE-HSV.Ul23:eGFP)^ia20^*, in which GFP expression is driven by 9X GRE tandem repeats. The GRE is shared by the activated homodimerized receptors of GCs, mineralocorticoids, progesterone, and androgens [170]. This line shows a significant increase in eGFP fluorescence, starting with a widespread pattern during early somitogenesis and becoming primarily concentrated in the brain and trunk muscles 24 h post-fertilization [171].

Recently, the Moro group generated a novel Nrf2/ARE pathway biosensor fish, *Tg(8xAORE:eGFP)^ia201^*, which exhibits a dynamic spatiotemporal expression profile during early developmental stages in skeletal muscle. Nrf2 is a basic leucine zipper transcription factor that binds to the promoter region of the antioxidant response element (ARE), inducing the coordinated upregulation of antioxidant and detoxification genes. The Nrf2 reporter system, harboring eight tandem-repeated ARE sequences (5′-GTGACAAAGCA-3′), is derived from mouse and rat Glutathione-S-Transferase Alpha (Gsta) promoters [172]. High levels of fluorescence were detected in the gut, in muscle fibers, and in the caudal region. Since 7 dpf (or at the early larval stage), fluorescence has been also detected in motorneurons and in the heart [173].

**Table 6:** Zebrafish reporter lines targeting in vivo signaling pathway activities involved in muscle development.

Line	Transgenic ID	Responsive Element (RE)	Signaling Pathway	Features	Ref.
*Tg(hsp70l:dnBmpr-GFP)^w30^*	ZDB-ALT-050503-2	BRE	BMP signaling	Heat shock (hsp70) promoter GFP expression	[164]
*Tg(BmpRE:mRFP)^cj100^*	ZDB-ALT-110705-4	BRE	BMP signaling	Membrane-bound red fluorescent protein (RFP)	[167,168]
*Tg(BmpRE-AAVmlp:eGFP)^mw29^*	ZDB-ALT-110308-1	BRE	BMP signaling	GFP expression	[166]
*Tg(BRE-AAVmlp:d2GFP)^mw30^*	ZDB-ALT-110310-1	BRE	BMP signaling	Destabilized d2GFP	[166]
*Tg(BRE-AAVmlp:dmKO2)^mw40^*	ZDB-ALT-110310-2	BRE	BMP signaling	Destabilized monomeric Kusabira-Orange (dmKO2)	[166]
*Tg(BMPRE:NLS-mCherry)^ia17^*	ZDB-ALT-130115-2	BRE	BMP signaling	Nuclear localization signal mCherry	[165]
*Tg(7xTCF-Xla.Siam:GFP)^ia4^*	ZDB-ALT-110113-1	TCF	Wnt signaling	GFP expression	[163]
*Tg(7xTCF-Xla.Siam:nlsmCherry)^ia5^*	ZDB-ALT-110113-2	TCF	Wnt signaling	Nuclear localization signal mCherry	[163]
*Tg(TOP:GFP)^w25^*	ZDB-ALT-020621-4	LEF binding sites	Wnt signaling	GFP expression	[161,162]
*Tg(6xCRE:eGFP)*		CRE	CREB signaling	Enhanced GFP expression	[169]
*Tg(4xHRE-TATA:eGFP)^ia21^*	ZDB-ALT-131030-1	HRE	Hypoxia signaling	Enhanced GFP expression	[156]
*Tg(12xgli-HSV.Ul23:GFP)^ia11^*	ZDB-ALT-120404-2	gli	Shh signaling	GFP expression	[159]
*Tg(8xGliBS:mCherry-NLS-Odc1)^st1002^*	ZDB-ALT-141030-2	gli	Shh signaling	Nuclear-localized fluorescent protein tagged with an ornithine decarboxylase-derived destabilizing peptide	[160]
* Tg(9xGCRE-HSV.Ul23:eGFP)^ia20^*	ZDB-ALT-130123-1	GRE	Glucocorticoid	Enhanced GFP expression	[171]
*Tg(8XAORE:GFP)^ia201^*	ZDB-ALT-211202-1	ARE	Nrf2/ARE	GFP expression	[173]

### 4.2. Zebrafish Reporter Lines Targeting the Skeletal Muscle

The zebrafish is a perfect vertebrate animal model to study the skeletal muscle *in vivo* both during development and disease progression. Furthermore, it is possible to create transgenic fish that may be used to label particular cell populations and monitor them over time, providing researchers with a rare chance to observe their live dynamics during development, damage, and regeneration. The different transgenic lines available for the live imaging of zebrafish muscle and stem/progenitor cells using confocal microscopes are provided in Table 7. Satellite cell markers that are well established and essential for muscle growth and regeneration include various transcription factors, including the paired homeobox genes *pax3* and *pax7*, the myogenic regulatory factor (MRF) *myf* gene, the myogenin gene (*myog*), and myogenic differentiation 1 (*myod*) [174].

Serger et al. generated the transgenic lines *TgBAC(pax7a:GFP)^i131^* and *TgBAC(pax3a:GFP)^i150^* expressing fluorescent proteins under the control of the *pax7a* or *pax3a* promoters, and they are able to mark progenitors and satellite cells that contribute to the embryonic development and postembryonic growth of skeletal muscle, as well as its repair and regeneration [175]. Transgenic lines for *myf5*, *myog*, and *myod* have also been created to track muscle development in real-time [174,175,176].

The specification of muscle cell types in the zebrafish embryo is greatly influenced by Shh signaling activity [177]. Maurya et al. [178] describe the identification of a minimal element of the *eng2a* gene sufficient to drive reporter gene expression in the muscle progenitors and medial fast fiber cells. 

The transgenic line *Tg(-10en2a:eGFP)^i233^* integrates repressive and activating signals from the BMP and Shh pathways, respectively, to restrict the expression of eGFP in muscle progenitors and medial fast fibers. 

The origin and mechanism of stem cell deployment or the clonal nature of muscle regeneration after injury could be more broadly assayed taking advantage of a muscle-specific lineage-tracing strategy using the zebrabow system, called “musclebow” [179]. The zebrabow line consists of a ubiquitous promoter driving three fluorophores (dTomato, mCerulean, and eYFP) in tandem and flanked by loxP sites. The construct’s default color is RFP (dTomato), but when Cre recombinase is present, the construct is stochastically deleted, allowing it to express either CFP (mCerulean) or YFP (eYFP). Since this excision is irreversible, the recombination event will be expressed in the cells’ offspring. As a result, cells and their offspring may be monitored over time. The achievement of muscle-specific recombination could be reached by crossing a muscle-specific CreERT2 line *Tg(msgn1: CreERT2)^pc9^* with the *Tg(ubi:Zebrabow)^a131^* line. Tamoxifen causes zebrabow rearrangements in all cells derived from the somites in these fish during embryogenesis because the mesogenin (*msgn1*) promoter is somite-specific [180]. These combinations of transgenes produce a “musclebow” fish, allowing the clonal relationships of muscle cells to be determined and resulting in individual fibers expressing distinctive color combinations. Newly differentiated fibers displayed a drift toward a single color (clonality) from the late larval stages until adulthood so that, in adults, entire sectors of each myotome have a uniform color. These findings show that multiple independent stem cells initially contribute randomly to fiber generation before a single stem cell clone eventually takes control of growth within individual myotomes over time due to repeated self-renewal events.

The transgenic line *Tg(mylz2:GFP)^gz8^* with the *mylz2* promoter is a specific molecular marker that monitors fast skeletal muscle development [181,182,183]. The GFP expression of the 1934-bp promoter transgenic lines closely mirrors the native *mylz2* mRNA expression pattern in both somitic and non-somitic muscles, including the fin, eye, jaw, and gill muscles. Moreover, the *Tg(tnnc1b:eGFP)^i305^* and *Tg(smyhc1:GFP)^i104^* transgenic lines faithfully recapitulate the development of slow skeletal muscle.

Two transgenic lines, *Tg(acta1:lifeact-GFP)^pc21^* and *Tg(acta1:mCherryCAAX)^pc22^*, are described by Berger et al. [184] to mark the myofibrils, sarcolemma, and t-tubules of the myofibers. In contrast, mCherry-CAAX integrates into the myofiber sarcolemma to fluorescently label the sarcolemma and t-tubules, while transgenic Lifeact-GFP highlights the striation of myofibrils. These two marker lines can be used together to examine and quantify the thickness of the myofibrils in individual live zebrafish myofibers [184,185]. In skeletal muscle disease, autophagy is a crucial cellular degradation system that transports cytoplasmic cargo to the lysosome [186]. Real-time autophagy monitoring is a crucial tool for understanding the formation of autophagosomes and determining the removal of proteins that are prone to aggregation. Two transgenic fluorescent zebrafish lines are used to monitor autophagy, one of which expresses RFP fused to LC3 *Tg(hsp70l:lamp1-RFP)^pd1064^* and the other of which expresses RFP fused to *lamp1b Tg(hsp70l:RFP-Rno.Map1lc3b)^pd1065^* under the control of the constitutive heat shock promoter [186,187].

Clark et al. created transgenic fluorescent zebrafish lines to create real-time and in vivo images of endosomes [188]. Based on the locations and roles of these endosome subtypes in zebrafish, scientists used Rab7 (late) to label late endosomes. These lines have been used to track autophagic activity in vivo and in real-time during development [186,187,188]. As previously described, zebrafish have many mitochondria in their skeletal muscles, which need high energy to contract and favor fish swimming. The balance of mitochondrial dynamics between fusion and fission keeps the web-shaped network in cells intact [189]. This balance is upset under specific physiological and pathological conditions, which leads to a change in mitochondrial morphology. The cytochrome c oxidase subunit VIIIA (*cox8a*) of zebrafish was cloned upstream of the eGFP gene by the Choi group [190]. Its expression was regulated by the widely used *Xenopus EF-1* promoter. This line allows for visualizing mitochondria in motor neurons, erythroid cells, photoreceptor cells, and skeletal muscles to study mitochondrial morphology. Breeding these zebrafish transgenic lines to disease model zebrafish and subsequently observing their mitochondrial morphology as the disease develops would be of help in exploring, in vivo, how changes in mitochondrial morphology can activate caspase-mediated apoptosis affecting the pathogenesis of muscle diseases [190,191].

**Table 7:** Zebrafish reporter lines targeting the skeletal muscle.

Line	Transgenic ID	Promoter	Cells Tagged	Features	Ref.
*Tg(myf5:YFP)^CLGY237^* *Tg(−80.0myf5: EGFP)^zf37^*	ZDB-ALT-150512-2 ZDB-ALT-070730-1	Myogenic factor 5	Quiescent and activated satellite cells	Yellow fluorescent protein Enhanced GFP expression	[176,192]
*TgBAC(pax7a:GFP)^i131^*	ZDB-ALT-111118-32	Paired box 7a	Muscle progenitors	GFP expression	[175]
*TgBAC(pax3a:GFP)^i150^*	ZDB-ALT-111118-34	Paired box 3a	Muscle progenitors	GFP expression	[175]
*Tg(myog:GFP)^pc27^*	ZDB-ALT-171003-14	Myogenin	Muscle progenitors	eGFP expression	[174]
*TgBAC(myod:GFP)^i124^*	ZDB-ALT-111118-35	Myogenic differentiation 1	Muscle progenitors	GFP expression	[175]
*Tg(-10en2a:EGFP)^i233^*	ZDB-ALT-110223-3	Engrailed homeobox 2a	Muscle progenitors and medial fast fibers	Enhanced GFP expression	[178]
*Tg(-2.2mylz2:GFP)^i135^* *Tg(mylz2:GFP)^gz8^* *Tg(mylpfa:mCherry)^cz3327^*	ZDB-ALT-081112-1 ZDB-ALT-080207-2 ZDB-ALT-130923-2	Myosin light chain, phosphorylatable, fast skeletal muscle	Fast skeletal muscle	GFP expression or mCherry	[181,182,183]
* TgPAC(prdm1a:EGFP)^i106^*	ZDB-ALT-080923-6	PR domain containing 1a, with ZNF domain	Primary and secondary slow fibers	Enhanced GFP expression	[193]
*Tg(tnnc1b:eGFP)^i305^*	ZDB-ALT-150723-4	Troponin C type 1b	Slow-twitch muscle fibers	Enhanced GFP expression	[146]
*Tg(Tru.Myhz1.1:EGFP)^kj100^*	ZDB-ALT-130823-1	Myosin, heavy polypeptide 1.1, skeletal muscle	Slow muscle-specific heavy chain	Enhanced GFP expression	[194]
*Tg(smyhc1:GFP)^i104^* *Tg(smyhc1:LY-Tomato)^oz29^*	ZDB-ALT-080923-4 ZDB-ALT-191024-1	Slow myosin heavy chain 1	Slow-twitch muscle fibers	GFP or tomato expression	[146,193,195]
*Tg(acta1: mCherryCAAX)^pc22^* *TgBAC(actc1b: GFP)^zf13: zf13Tg^* *Tg(acta1:lifeact-GFP)^pc21^*	ZDB-ALT-150224-2 ZDB-ALT-060221-2 ZDB-ALT-150224-1	Actin alpha cardiac muscle 1b	Myofibrils, sarcolemma, and t-tubules of the myofibers	Membrane-tethered mCherry or GFP	[184,185]
*Tg(ubi: zebrabow-M)^a131^* *Tg(msgn1: CreERT2)^pc9^*	ZDB-ALT-130816-2 ZDB-ALT-141117-7	Mesogenin 1	Muscle progenitors	dTomato, mCerulean and eYFP)	[196,197]
* Tg(hsp70l:lamp1-RFP)^pd1064^*	ZDB-ALT-130409-7	Lysosomal-associated membrane protein 1b	Skeletal muscle lysosomes	RFP expression	[186,187]
* Tg(hsp70l:RFP-Rno.Map1lc3b)^pd1065^*	ZDB-ALT-130410-5	Microtubule-associated protein 1 light chain 3 beta	Skeletal muscle lysosomes	RFP expression	[186,187]
* Tg(h2ax:EGFP-rab7a)^mw7^*	ZDB-ALT-111017-6	RAB7a, member RAS oncogene family	Skeletal muscle Late endosome	Enhanced GFP expression	[188]
*Tg(Xla.Eef1a1:mlsEGFP) ^cms1^*	ZDB-ALT-090309-2	Cytochrome c oxidase subunit 8A	Mitochondria	Enhanced GFP expression	[190,191]

## 5. Zebrafish Models of MDs Used for Drug Screening

Nonmammalian animal models, such as *Caenorabditis elegans*, *Drosophyla melanogaster*, and *Danio rerio*, are particularly attractive in conducting cost-effective, high-throughput drug screenings. This is because of the ease of genetic manipulation, high offspring numbers, and low ethical concerns compared with existing mammalian models such as mdx mice and Golden Retriever dogs [198]. The major issue of mdx mice, which is the most used animal model for DMD, is the mild clinical presentation due to compensatory phenomena [199,200]. For that reason, several mdx mouse derivatives have been developed to have a disease phenotype more similar to humans and Golden Retrievers [201,202]. Among nonmammalian models, zebrafish models of myopathies show severe disease phenotypes and have been extensively used for high-throughput drug screening. A second advantage of zebrafish models is that embryos can easily absorb drugs dissolved in embryo water, which is useful in assessing the effects during the first stages of disease development [203]. Several lead compounds able to ameliorate major disease symptoms, such as muscle fibrosis, inflammation, dysregulation of calcium homeostasis, oxidative stress, and mitochondrial dysfunction, have been discovered by using DMD *sapje* and *sapje-like* zebrafish models generated with ENU mutagenesis (see Table 2) [117,118,137,198,204,205,206,207,208], LGMD2I zebrafish models harboring the *fkrp* mutation (see Table 3) [76], the zebrafish transgenic model of myotonic dystrophy harboring multiple CUG repetitions in its genome [209], zebrafish models of collagen VI deficiency congenital myopathies (Table 1) [119,210,211], and *candyfloss* zebrafish models [33].

## 6. Conclusions

Zebrafish mutant lines, transgenic fluorescent biosensors, and phenotyping assays that have been extensively used in biomedical research to study muscle development and perform high-throughput drug screening were summarized in this review. All these tools are essential to understanding the molecular mechanisms underlying both the pathogenesis of muscular dystrophies and skeletal muscle development and organization. Muscle-specific transgenic zebrafish biosensors are a powerful tool to track the cell dynamics in a living animal at the level of a single muscle fiber at the embryonic or larval stages. Thanks to the advances in new technologies, such as CRISPR/Cas9, we will be able to both generate even more specific zebrafish lines mimicking human muscular dystrophies and to progress toward precision medicine by repairing muscle defects in patients showing specific mutations in specific muscle genes.

## Figures and Tables

**Table 1:** Zebrafish models for MDs linked to the basal membrane and ECM proteins.

Gene Symbol	Protein	Technique	Ref.
*lama2*	Laminin-α2	ENU screen ENU screen	[8] [38]
*col6a1*	Collagen, type VI, alpha 1	CRISPR/Cas9 TALEN mut. MO	[44] [45] [46]
*col6a3*	Collagen, type VI, alpha 3	MO	[46]
*col22a1*	Collagen, type XXII, alpha 1	MO CRISPR/Cas9	[43] [47]

**Table 2:** Zebrafish models for MDs linked to cytosolic proteins.

Gene Symbol	Protein	Technique	Ref.
*dmd*	Dystrophin	Genetic screen ENU screen MO	[49] [52] [62]
*dux4*	Double homeobox protein 4	mRNA injection Tol2 transposon system Tol2 transposon system	[53] [54] [55]
*cavin1a/b*	Caveolae-associated protein 1	MO	[63]
*hnrnpdl*	Heterogeneous nuclear ribonucleoprotein D-like	MO	[64]
*mbnl1/2/3*	Muscleblind-like splicing regulator	CRISPR/Cas9 mRNA injection	[56] [58]
*vcp*	Valosin containing protein	CRISPR/Cas9 MO	[59] [60] [61]
*mbnl2*	Muscleblind-like splicing regulator 2	MO	[57]
*tcap*	Telethonin	MO	[65]
*ttn*	Titin	MO	[66]
*bves*	Blood vessel epicardial substance	MO; TALEN mut.	[67]
*inpp5ka/b*	Inositol polyphosphate 5-phosphatase K	MO	[68,69]
*craa/b*	Cristallin	MO	[61]
*bag3*	BCL2 associated athanogene 3	MO	[61,70]
*fhl1a/b*	Four and a half LIM domains 1	MO	[61,71]
*ldb3*	LIM domain binding 3	MO	[61,72]

**Table 3:** Zebrafish models for MDs linked to dystroglycan and α-DG glycosylation-related proteins.

Gene Symbol	Protein	Technique	Ref.
*Primary dystroglycanopathy*			
*dag1*	Dystroglycan	MO ENU screen	[48] [75]
*Secondary dystroglycanopathies*			
*pomt1*	O-mannosyl transferase	MO	[78]
*pomt*2	O-mannosyl transferase	MO	[78]
*pomgnt2*	O-mannose β-1,4-Nacetylglucosaminyltransferase	MO	[79]
*fktn*	Fukutin	MO MO	[80] [81]
*fkrp*	Fukutin-related protein	TALEN mut.; Tol2 transposon system Zinc finger nuclease KO; CRISPR/Cas9 MO MO MO MO	[76] [77] [80] [81] [82] [83]
*ispd*	CDP-ribitol pyrophosphorylase	MO	[84]
*rxylt1*	β−1,4-xylosyl transferase	MO	[85]
*b3galnt2*	β−1,3-N-acetylgalactosaminyltransferase	MO	[86]
*b4gat1*	β−1,4-glucuronyltransferase	MO	[87]
*dpm1*	Dolichol–phosphate mannose synthase	MO	[88,89]
*dpm2*	Dolichol–phosphate mannose synthase	MO	[89]
*dpm3*	Dolichol–phosphate mannose synthase	MO	[89]
*pomk*	O-mannose kinase	MO	[90]
*gmppb*	GDP-mannose pyrophosphorylase	MO	[91]

**Table 4:** Zebrafish models for MDs linked to the nuclear envelope and cytoskeleton proteins.

Gene Symbol	Protein	Technique	Ref.
*desma/b*	Desmin	MO ENU screen MO	[61] [94] [96]
*lmna*	Lamin-A/C	CRISPR/Cas9 mut. MO	[98] [101]
*myot*	Myotilin	MO	[61]
*dnajb6a/b*	dnaJ homolog subfamily B member 6	MO MO mRNA injection	[61] [102] [103]
*pleca/b*	Plectin	MO	[61]
*flnca/b*	Filamin C	ENU screen, MO; Genetic screen	[99] [100]
*sgcd*	δ-sarcoglycan	MO	[104,105]
*dmpk*	Myotonic dystrophy protein kinase	mRNA injection	[58]

**Table 5:** Zebrafish models for MDs linked to membrane proteins.

Gene Symbol	Protein	Technique	Ref.
*itga7*	Integrin-α7	MO	[109]
*cav3*	Caveolin-3	Tol2 transposon system MO; mRNA injection	[63] [108]
*dysf*	Dysferlin	MO	[110,111]

## Data Availability

Non applicable.
